# Exploring the Dynamic Range of the Kinetic Exclusion Assay in Characterizing Antigen-Antibody Interactions

**DOI:** 10.1371/journal.pone.0036261

**Published:** 2012-04-30

**Authors:** Christine Bee, Yasmina N. Abdiche, Donna M. Stone, Sierra Collier, Kevin C. Lindquist, Alanna C. Pinkerton, Jaume Pons, Arvind Rajpal

**Affiliations:** Rinat, Pfizer Inc., South San Francisco, California, United States of America; Griffith University, Australia

## Abstract

Therapeutic antibodies are often engineered or selected to have high on-target binding affinities that can be challenging to determine precisely by most biophysical methods. Here, we explore the dynamic range of the kinetic exclusion assay (KinExA) by exploiting the interactions of an anti-DKK antibody with a panel of DKK antigens as a model system. By tailoring the KinExA to each studied antigen, we obtained apparent equilibrium dissociation constants (K_D_ values) spanning six orders of magnitude, from approximately 100 fM to 100 nM. Using a previously calibrated antibody concentration and working in a suitable concentration range, we show that a single experiment can yield accurate and precise values for both the apparent K_D_ and the apparent active concentration of the antigen, thereby increasing the information content of an assay and decreasing sample consumption. Orthogonal measurements obtained on Biacore and Octet label-free biosensor platforms further validated our KinExA-derived affinity and active concentration determinations. We obtained excellent agreement in the apparent affinities obtained across platforms and within the KinExA method irrespective of the assay orientation employed or the purity of the recombinant or native antigens.

## Introduction

The kinetic exclusion assay (KinExA) is a solution-based method to determine the concentrations of interacting binding partners and the equilibrium dissociation constants (K_D_) of biomolecular interactions, typically in the pM to low nM range. When applied to the study of antigen/antibody interactions, the antigen is typically titrated into a constant concentration of antibody binding sites, the samples are allowed to equilibrate, and then drawn quickly through a flow cell where free antibody binding sites are captured on antigen-coated beads, while the antigen-saturated antibody complex is washed away. The bead-captured antibody is then detected with a fluorescently labeled anti-species antibody [1]. We aimed to explore the dynamic range, versatility, and the precision of this technique with a suitable panel of antigen/antibody interactions. We therefore sought out a monoclonal antibody (mAb), named DS4, that was raised against human Dickkopf protein 1 (DKK1) and cross-reacted with other members of the DKK family with disparate affinities. Not only did these reagents provide us with a model interaction system, but DKK proteins have gained growing interest as therapeutic targets due to their implication in bone disease, cancer, and Alzheimer's disease [2]. The human DKK protein family consists of four members (DKK1, DKK2, DKK3, and DKK4) that each contain two conserved cysteine-rich domains [3] and are monomeric, secreted glycoproteins with molecular weights of approximately 25 kDa that regulate Wnt signaling in different ways [2], [4]. The solubility and elevated serum levels of DKK proteins during some disease states make them attractive targets for antibody therapy, notably DKK1, which is the most extensively characterized.

DKK proteins present practical challenges to biosensor-based interaction analysis for a variety of reasons: purified preparations of some DKK proteins were only available in limited quantity; other DKK proteins were only available in unpurified form in conditioned media; being glycoproteins they may be differentially glycosylated in recombinant and native forms; and they have a high theoretical net positive charge (ranging from +10 to +22) at neutral pH (except DKK3, which has a theoretical net charge of −30). Furthermore, our model mAb DS4 bound DKK1 with an extremely slow dissociation rate constant approaching the resolution limit of direct detection on Biacore biosensors [5], [6]. The KinExA measurements outlined in this study for DS4 binding a multi-species panel of DKK proteins spanned a K_D_ range from approximately 100 fM to 100 nM and, where appropriate, we corroborated them with orthogonal measurements on label-free biosensor platforms. We present methods to reduce antigen consumption and measure accurate and precise affinities of a variety of DKK proteins that differed in their available quantity and quality.

## Results

### The KinExA offers two different assay orientations for K_D_ determination

**Figure 1:**
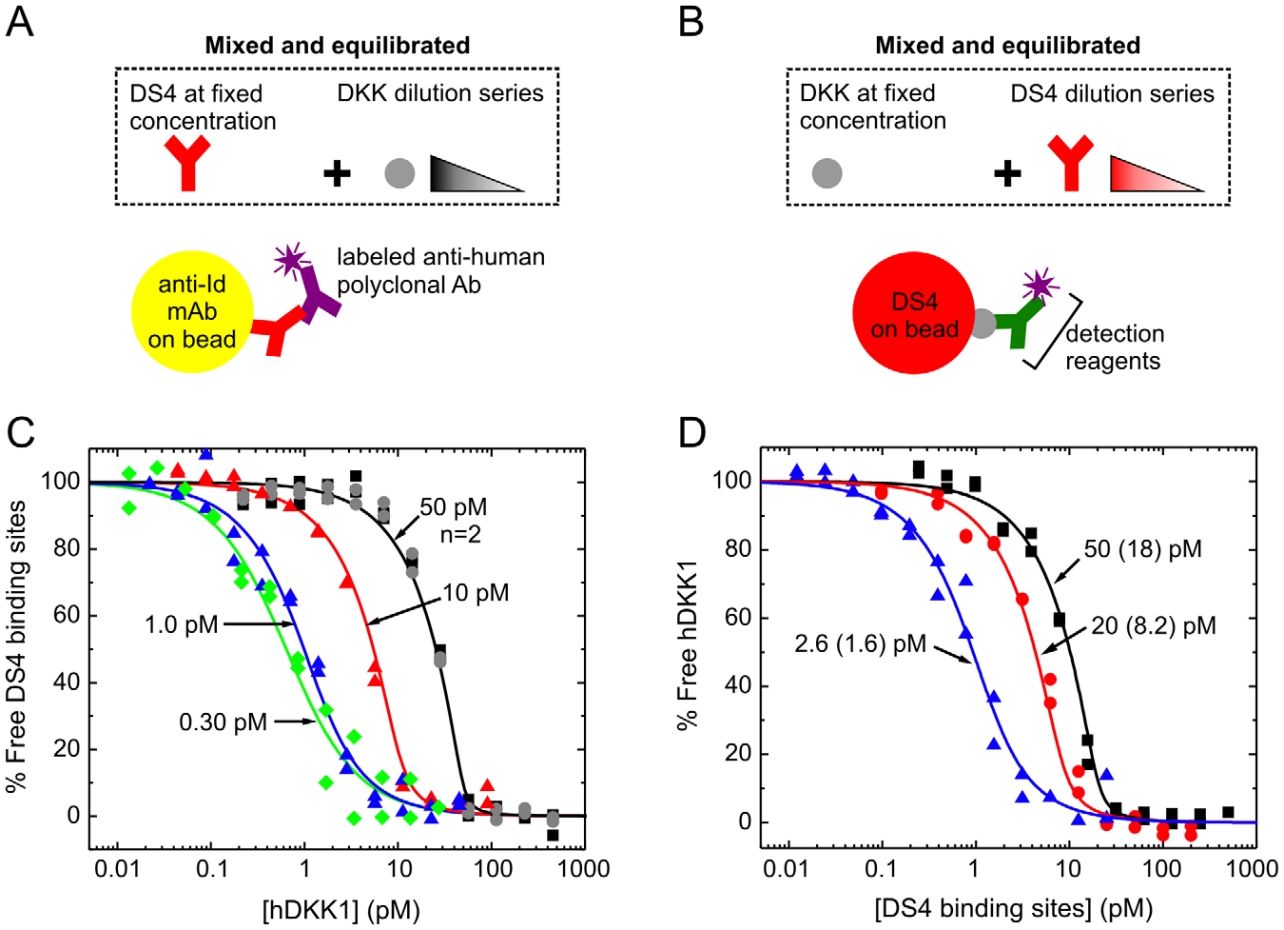
DKK1/DS4 interaction studied in the fixed antibody and fixed antigen assay orientations on the KinExA. (A) In the fixed antibody orientation, a series of samples is prepared by titrating DKK into a fixed concentration of antibody binding sites. After sample equilibration, free antibody binding sites are captured on beads and detected by a fluorescently labeled anti-species antibody. In our modified KinExA method, the beads are coated with a murine anti-idiotypic mAb instead of antigen. (B) In the fixed antigen orientation, a series of samples is prepared by titrating the antibody into a fixed DKK concentration. Free DKK in equilibrated samples is captured on antibody-coated beads and detected with a customized sandwiching mAb that is fluorescently-labeled (one step detection) or unlabeled and followed by a fluorescently-labeled reagent (two step detection); see Materials and Methods. (C) Global analysis of DS4's interaction with human DKK1 in the fixed antibody orientation. The “unknown ligand” model in the KinExA software automatically corrects the concentration of the titrated component with the best fit for its apparent activity, so that the x-axis shows the antigen's active concentration, rather than its nominal concentration. (D) Global analysis of DS4's interaction with human DKK1 in the fixed antigen orientation. For both panels C and D, the nominal concentration of the fixed binding partner is indicated per titration curve; in panel D, the best fit binding site concentration is indicated in parentheses. The apparent K_D_ values for panels C and D were 0.49 pM and 0.42 pM, respectively (see Table 1).

Figure 1 shows the two different assay orientations that we employed to measure the affinity of DS4 binding a panel of eight DKK proteins, which included human DKK1 and its homologs and orthologs. The “fixed antibody” KinExA orientation (Figure 1A) requires beads to be coated with an interaction partner that specifically binds the free antibody binding sites but not the antigen-saturated antibody complex [7], [8]. Typically, the antigen is adsorption-coated on “hard” beads (e.g., polymethylmethacrylate, PMMA [1]) or covalently coupled onto functionalized “hard” (e.g., NHS-PMMA [9]) or “soft” beads (e.g., azlactone [10] or NHS-activated sepharose [5]). Immobilization requirements reported in the literature range from 10 µg [9] to 100 µg [11] antigen per experiment where titration curves are typically analyzed in duplicate. Purified DKK1 and its homologs were not available to us in the quantities required for bead coating. We therefore modified the fixed antibody method by using a murine anti-idiotypic (anti-Id) mAb as the bead-immobilized capture reagent (Figure 1A). The specific anti-Id used was raised via hybridoma technology against DS4 and was selected because it only bound to free antibody binding sites and not to the antigen-saturated antibody complex. A fraction of free DS4 binding sites present in each sample was captured on the beads and detected with a fluorescently-labeled polyclonal anti-human antibody with minimal cross-reactivity to murine antibodies.

Figure 1B presents an alternate assay orientation [12] where a fixed concentration of antigen is titrated with DS4 and a portion of the free antigen in these mixtures is detected on DS4-coated beads; we refer to this assay format as the “fixed antigen” orientation. Unlike the fixed antibody orientation, where the same secondary detection reagent is used regardless of the antigen being studied, the fixed antigen assay requires a customized detection strategy for every antigen. Therefore, as we explored the multi-species panel of DKK1 and its homologs, we tailored the detection reagents to each antigen in this assay format.

### The two KinExA orientations yielded equivalent K_D_ values

We used the fixed antibody KinExA orientation (Figure 1A) to determine the affinity of DS4 towards purified recombinant human DKK1. To increase the precision of the K_D_ determination, we performed multiple experiments, in which we titrated the antigen into a constant antibody binding site concentration and varied the antibody's concentration from experiment to experiment. Fixing the antibody at a concentration that is at or below the K_D_ will typically contain sufficient information to quantify the K_D_ value and give a shallow K_D_-controlled titration curve, while higher antibody concentrations will enable the determination of the active concentration of one interaction partner (antibody or antigen) relative to the other. We refer to the “activity” of the antigen as the percentage of its nominal concentration that can exactly titrate out an equimolar concentration of previously calibrated (i.e., “known”) antibody binding sites, when inhibition is examined at high enough concentrations to promote a steep titration curve (see Materials and Methods). Such curves are often referred to as “receptor-controlled” [7] in the KinExA literature, but here we prefer the term “stoichiometry-controlled”, to avoid confusion when describing interactions that are not ligand/receptor interactions. In studying human DKK1, we combined information from five curves, which included both K_D_-controlled curves and stoichiometry-controlled curves, by fitting them globally to give a robust estimate of both the apparent K_D_ and the apparent active antigen concentration. In this way, we determined that DS4 had an apparent K_D_ of 490 fM for purified recombinant human DKK1 (Figure 1C) and that the latter was 91% active.

To determine whether the fixed antibody and fixed antigen assay orientations yielded similar K_D_ values, we also analyzed the interaction of purified recombinant human DKK1 and DS4 in the fixed antigen orientation (Figure 1B). We found that a single-step detection strategy, using DyLight-labeled anti-human DKK1 antibody, not only decreased the assay run time but lowered the unspecific binding signal in the flow cell. By varying DKK1's concentration from experiment to experiment we generated multiple curves and fit them globally. We thus found that the best fit K_D_ values determined from the fixed antibody and fixed antigen assay orientations were identical within the 95% confidence limits of each global fit (compare Figures 1C and 1D and see Table 1).

**Table 1:** KinExA affinity measurements.

Antigen	Source	Fixed binding partner (nominal, pM)^b^	% activity^c^	Apparent K_D_ (pM)^c^	n^g^
Human DKK1	Purified	antibody (0.3, 1, 10, 50)	91 (68–120)	0.49 (0.21–0.96)	5
		antigen (3, 10, 50)	39 (28–52)	0.42 (0.13–0.91)	3
	Unpurified^a^ (Hep3B cells)	antigen (1/20, 1/1000)	n.a.^d^	0.16 (0.048–0.34)	2
Mouse DKK1	Purified	antibody (60, 2, 1)	25 (19–36)	< 0.3	3
Rat DKK1	Purified	antibody (50, 5, 0.5)	50 (42–59)	0.62 (0.43–0.92)	3
Human DKK2	Unpurified (293F cells)	antigen (1/3)	n.a.	110,000 (56,000–130,000)	1
Cyno DKK2	Unpurified (293F cells)	antigen (1/3)	n.a.	120,000^e^ (82,000–160,000)	1
				170,000^f^ (93,000–240,000)	1
Human DKK4	Purified	antibody (30, 5)	33(27–43)	3.5 (2.2–5.5)	2
		antigen (40)	45 (25–63)	2.9 (1.1–5.8)	1
Rhesus DKK4	Unpurified (293F cells)	antibody (40, 10)	n.a.	2.8 (0.9–7.7)	2
Mouse DKK4	Purified	antibody (10, 30)	47 (35–74)	5.1 (2.6–10)	12
		antibody (5, 30)	46 (37–59)	4.6 (3.1–6.9)	2

a All antigens were recombinant, except when using Hep3B cells, which express native human DKK1.

b Nominal concentration in pM or dilution factor (1/x) for unpurified samples.

c Best fit (and the 95% confidence interval) as determined in the KinExA analysis software.

d Not applicable.

e 0.3 mL/min sample flow rate.

f 1.2 mL/min sample flow rate.

g Number of independent experiments analyzed globally where each titration curve represents duplicate measurements.

Having established that both assay orientations yield equivalent results, we determined the affinities of DS4 towards a multi-species panel of purified recombinant DKK1 proteins using the fixed antibody assay orientation because this allowed us to apply the same detection strategy to all the antigens. Within the 95% confidence limits of each fit, the antibody showed the same affinity towards all DKK1 orthologs studied (Table 1).

We also studied the cross-reactivity of DS4 to purified recombinant human DKK4 via both the fixed antibody and fixed antigen assay orientations. These experiments yielded best fit K_D_ values in the single digit picomolar range that were identical within the 95% confidence interval of each experiment regardless of the assay orientation used (see Table 1). Similar results were obtained for the interaction of purified recombinant mouse DKK4 with DS4.

### A single experiment can yield with good precision both an accurate apparent K_D_ and an accurate antigen activity when one binding partner is fixed at a suitable concentration

To assess the reproducibility, precision, and accuracy of our KinExA-based K_D_ determinations, we chose the mouse DKK4/DS4 interaction as a model system due to the practical conveniences of studying a low picomolar affinity rather than a sub-picomolar affinity (discussed later) and the availability of a high quality purified preparation of the mouse DKK4.

**Figure 2:**
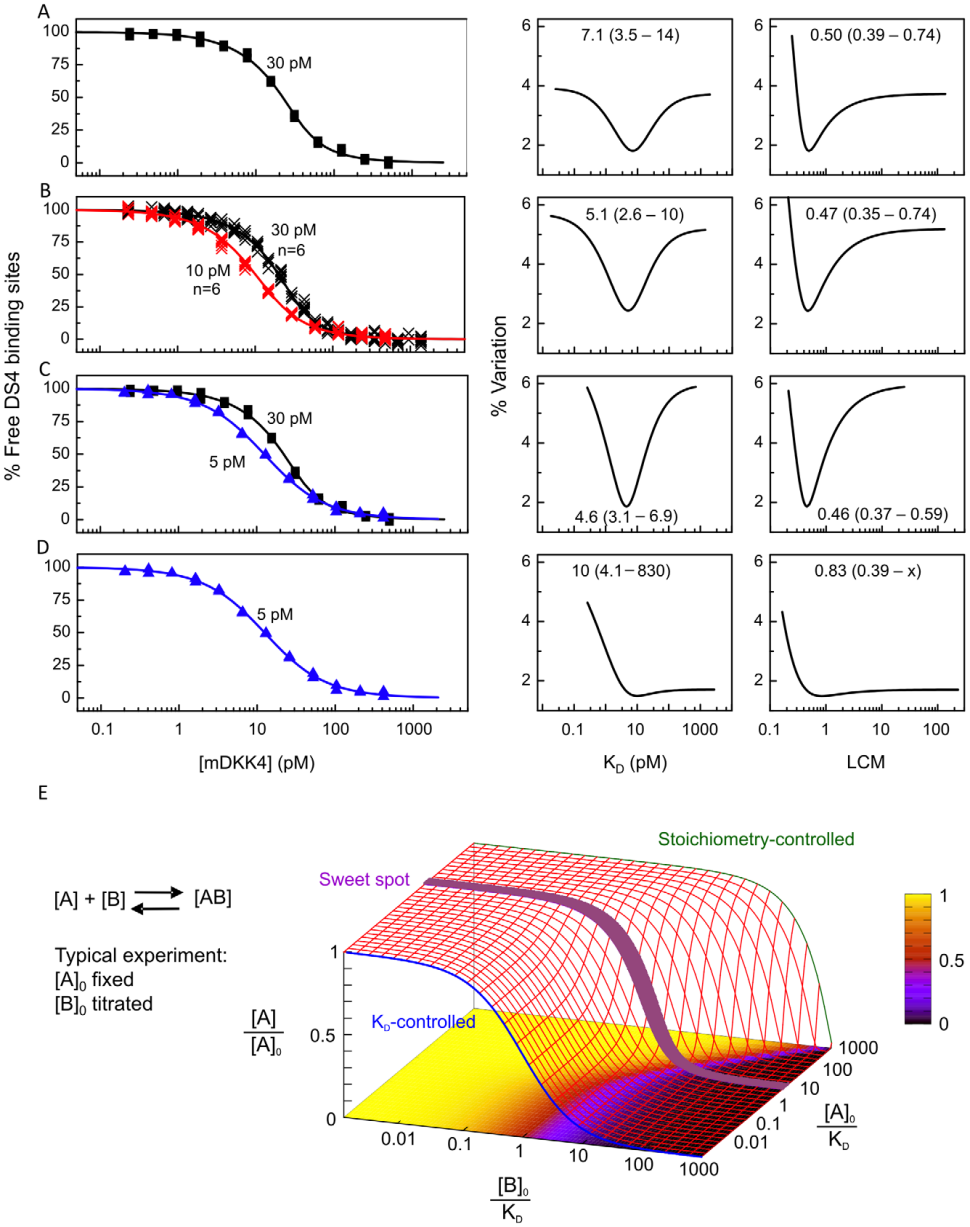
Mouse DKK4/DS4 interaction studied at different fixed antibody concentrations. Left panel - overlay plots of the measured data and the theoretical best fit titration curve resulting from a four-parameter equation (as defined in the KinExA software), center panel - error curves for the best fit K_D_, and right panel - error curves for the best fit antigen activity correction factor (referred to in the KinExA software as the ligand concentration multiplier, LCM). An LCM of 0.5 indicates that the antigen is 50% active. (A) Single experiment with 30 pM antibody binding sites. (B) Global analysis of twelve independent experiments that incorporated six curves each at 10 pM and 30 pM antibody binding sites. (C) Dual-curve analysis for 30 pM and 5 pM antibody binding sites and (D) individual analysis of the 5 pM curve. (E) Contour plot for the bimolecular binding equation that shows the theoretical titration curves obtained for different ratios of the concentration of the fixed binding partner over the K_D_, i.e., the [A]_0_/K_D_ ratio. The empirical sweet spot ratio is indicated in violet and the bottom plane shows a color-coded projection of the fraction of unbound [A] ([A]/[A]_0_) as a function of [B]_0_/K_D_ and [A]_0_/K_D_. The model used to create this figure is: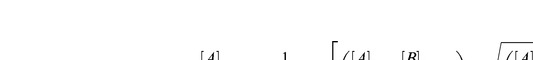.

We tested purified recombinant mouse DKK4 in the fixed antibody assay orientation, as shown in Figure 2A for an experiment that used an antibody binding site concentration of 30 pM. Upon repeating the measurement six independent times we obtained a mean apparent K_D_ of 6.9 pM with a standard deviation of 0.6 pM, indicating that the experiment was highly reproducible. We performed six further experiments at an antibody binding site concentration of 10 pM and fit all twelve curves globally as shown in Figure 2B. The center and right panels in Figure 2 show error plots for the K_D_ and the activity correction factor (known as the “ligand concentration multiplier”, LCM, in the KinExA software), respectively. The error plots show the percent variation in a given parameter (either K_D_ or LCM) by fixing it and floating the other parameter in a least squares fit [7]. The optimum K_D_ (or LCM) corresponds to the minimum in the respective graph [1]. Globally fitting two curves obtained using 30 pM and 5 pM antibody binding sites increased the precision of the K_D_ determination by about twofold compared with that obtained at 30 pM alone, irrespective of the number of 30 pM curves included in the global analysis (compare Figure 2A to 2C); the K_D_ determined from the 5 pM curve alone had broad, ill-defined bounds (Figure 2D). However, analyzing any one of the six experiments performed with a fixed antibody concentration of 30 pM allowed both floated parameters, namely the apparent K_D_ and the antigen's apparent activity, to be determined accurately, as judged by the best fit values falling within the 95% confidence limits of an extensive n-curve analysis (compare the error curves of Figure 2A to those of Figure 2B or 2C), and with good precision, as judged by the narrow bounds that spanned a range less than twofold the parameter value (see error curves of Figure 2A). We found that this information-rich curve could be obtained if one works at a suitable ratio of the concentration of the fixed binding partner over the best fit K_D_. This ratio is reported in the KinExA software and we refer to it as the sweet spot ratio (which was typically 4 to 6 in our experiments) when both floated parameters can be determined accurately and with good precision in a single experiment. Figure 2E indicates the location of this empirically-determined sweet spot ratio in the context of a three-dimensional contour plot of the bimolecular binding equation and shows the shapes of the theoretical titration curves obtained at different ratios of the concentration of the fixed binding partner relative to the K_D_.

### Unpurified antigen that was available at concentrations several orders of magnitude above K_D_ was amenable to study in the fixed antibody orientation

**Figure 3:**
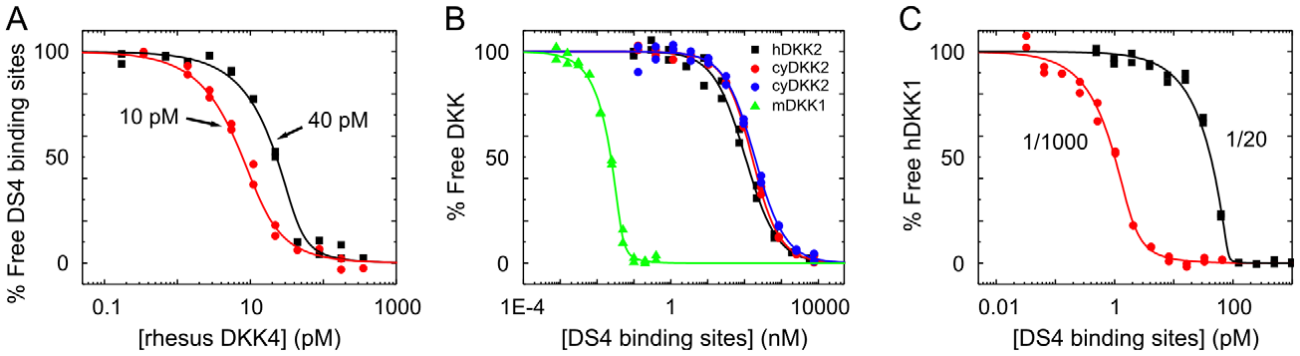
Affinity determinations for crude antigens from conditioned media. (A) Dual-curve analysis in the fixed antibody assay orientation of recombinant rhesus DKK4 in conditioned media of 293F cells. (B) Overlay plot of the curves obtained in the fixed antigen assay orientation using recombinant human DKK2 (black squares) and cyno DKK2 (red circles) from conditioned media of 293F cells, each diluted threefold with running buffer. The blue curve shows the cyno DKK2 assay performed at a fourfold higher flow rate and the green curve represents a control titration of purified mouse DKK1 spiked into a threefold dilution of media from untransfected cells. (C) Dual-curve analysis in the fixed antigen assay orientation of native human DKK1 in the media of a Hepatocarcinoma cell line, diluted 1000-fold (red) and 20-fold (black) with running buffer. The apparent K_D_ values were (A) 2.1 pM – rhesus DKK4, (B) 110 nM – human DKK2; 120 nM (red) and 170 nM (blue) – cyno DKK2, and (C) 0.16 pM – human DKK1 (see Table 1).

Recombinant rhesus DKK4 was heterologously expressed in-house by transfection of 293F cells. Since its concentration in conditioned media was estimated to be approximately 10 µg/mL by ELISA (data not shown), we used the crude media as the source of the antigen and determined its affinity for DS4 using the fixed antibody assay orientation. We first aimed for a stoichiometry-controlled curve to determine the active concentration of rhesus DKK4 in the media. Instead of using a known antigen concentration, we diluted the conditioned media 1000-fold into sample buffer containing 40 pM antibody binding sites and used this sample to represent the top concentration of an antigen dilution series into a background of 40 pM antibody binding sites. We determined the rhesus DKK4 concentration to be 310 pM in the 1000-fold diluted media, which corresponded to 310 nM (or 8 µg/mL) in the undiluted media; this was in close agreement with the estimation from ELISA. With this knowledge, we obtained a K_D_-controlled curve by fixing the antibody binding site concentration at 10 pM and titrating it with an appropriate concentration range of the antigen. A global fit of both curves (Figure 3A) yielded an apparent K_D_ of 2.8 (0.9–7.7) pM.

### Unpurified antigen that was available at concentrations near or below K_D_ could be studied via the fixed antigen orientation

To investigate whether the KinExA could accurately determine the affinity of a nanomolar binder, we studied DS4's cross-reaction with DKK2. Attempts to purify human and cynomolgus monkey (cyno) DKK2 were unsuccessful and so they were available only in unpurified form and at low concentrations in crude conditioned media. Thus, we titrated DS4 into conditioned media that was diluted threefold in running buffer. More than 10,000-fold higher concentrations of DS4, relative to those used for DKK1, were needed to saturate DKK2 at the same sample volume, consistent with DKK2 binding with a significantly weaker affinity than DKK1. The apparent K_D_ values for the interaction of DS4 with human and cyno DKK2 were determined to be 110 (56–130) nM and 120 (82–160) nM respectively (Figure 3B). We could not quantify the DKK2 in the conditioned media samples because the curve was K_D_-controlled, suggesting that the threefold diluted conditioned media contained ≤100 nM DKK2. Working with undiluted media to estimate the antigen's active concentration would have required the use of at least 30 mg of DS4 and thus was not further explored.

To verify that the KinExA method could return an accurate affinity for a relatively weak affinity interaction without perturbing equilibrium, we repeated the cyno DKK2 experiments at a fourfold faster flow rate (i.e., 1.2 mL/min compared to 0.3 mL/min) to shorten the exposure time of the equilibrated sample with the bead. If the equilibrium was significantly perturbed over the bead exposure times used in our experiments, we would expect the apparent K_D_ to differ between the two flow rates; however, the data we obtained at different flow rates were superimposable and the apparent K_D_ values were identical within the error margins (Table 1).

In order to exclude possible artifacts that may be introduced by the complex composition of the cell culture media, we validated the assay format using a purified His-tagged DKK with a known K_D_ in a background of conditioned media in which the cells were not transfected with an expression vector (i.e., negative control media). As the model antigen, we chose purified recombinant mouse DKK1 because it had the same polyhistidine -tag as our human and cyno DKK2 constructs. Titrating DS4 into a nominal concentration of 44 pM mouse DKK1 prepared in the negative control media that was diluted threefold in sample buffer resulted in a sharp, stoichiometry-controlled curve several orders of magnitude below DS4 concentrations necessary to titrate DKK2 (Figure 3B), consistent with DKK1 having a much tighter affinity than DKK2 for DS4.

### Native human DKK1 available in conditioned media of a carcinoma cell line was amenable to a KinExA analysis

Rathanaswami et al. recently described the application of the fixed antigen KinExA method to measure the active concentration of an unpurified native antigen (i.e., human IL13) contained in conditioned media and its affinity towards different antibodies [10]. We employed this method to determine the affinity of DS4 towards unpurified native human DKK1 contained in conditioned media from the hepatic carcinoma cell line Hep3B. We generated both stoichiometry-controlled and K_D_-controlled curves by titrating the antibody into media that was diluted 20-fold or 1000-fold respectively in sample buffer. A global fit of these two curves gave an apparent K_D_ of 160 (50–340) fM (Figure 3C). The concentration of native human DKK1 in the undiluted media was determined to be 1.7 (1.4–2.0) nM (or 44 ng/mL). This corroborated an ELISA estimate of >30 ng/mL (data not shown).

### Active antigen concentrations were corroborated via stoichiometry-controlled titrations on an Octet biosensor

**Figure 4:**
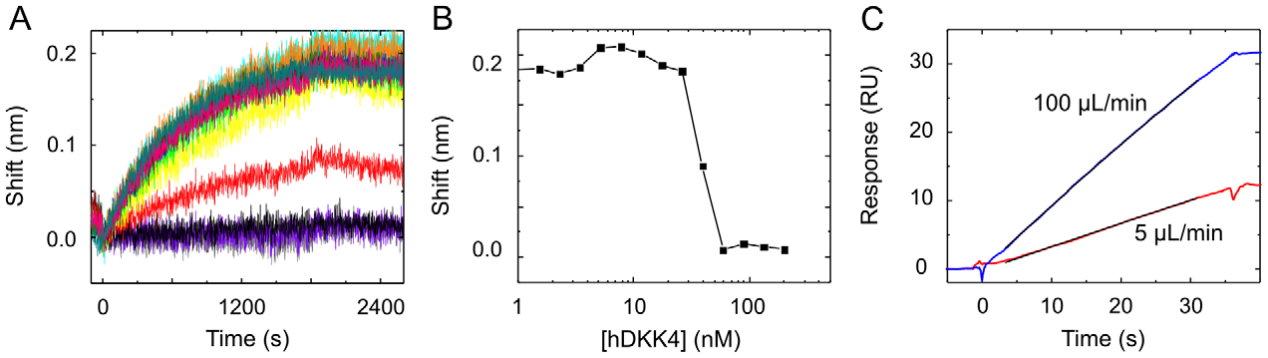
Determination of active antigen concentrations using complementary label-free methods. (A) Titration-based Octet measurement obtained over immobilized anti-Id mAb for samples containing 1 nM DS4 binding sites titrated with a purified preparation of carrier-free human DKK4. (B) Sharp inhibition curve obtained from the data shown in panel A showing that a nominal concentration of 50 nM human DKK4 was needed to exactly titrate out 1 nM DS4 (corresponding to an antigen activity of 2%). (C) CFCA data collected on the Biacore for a nominal concentration of 0.1 µg/mL human DKK1 flowed at 100 µL/min (blue) and 5 µL/min (red) over a high capacity of immobilized DS4. The curve fit is shown in black.

In order to validate our KinExA experiments using an orthogonal label-free method, we performed Biacore analyses on a suitable panel of DKK1, DKK2, and DKK4 proteins, thus covering the full affinity range studied on the KinExA. In these assays, purified recombinant antigens were flowed over immobilized DS4. Since the accuracy of the observed k_a_ (and thus the observed K_D_) depends on the accuracy of the antigen's active concentration, the observed k_a_ from our Biacore experiments had to be corrected for any difference between the nominal and active concentration, which required a separate assay for every antigen studied (Figure 4). We thus determined the active concentration of each antigen using a solution competition assay on an Octet biosensor. The Octet is a label-free interferometry-based technology equipped with disposable fiber-optic sensor tips that measures the change in optical thickness that occurs upon the real time binding of a solution partner to an immobilized partner on a sensor tip. Assuming that DS4 was fully active, we determined the activities of various DKK proteins by conducting stoichiometry-controlled titrations on the Octet and used these values to correct the apparent k_a_ values obtained in our Biacore experiments (Table 2). The Octet experiments revealed that some commercially available preparations of purified antigens were mostly inactive, as shown in Figures 4A and 4B, i.e., a carrier-free preparation of human DKK4 appeared only 2% active. Since antigens that were lyophilized from BSA-containing buffers typically appeared more active than carrier-free formulations, we opted to use the former where available for all our affinity determinations.

**Table 2:** Biacore kinetic and affinity determinations on purified, recombinant antigens.

Antigen	% activity^a^	Apparent k_a_ (M^−1^s^−1^)^d^	Apparent k_d_ (s^−1^)	Apparent K_D_ (pM)	Apparent K_D_ by KinExA (pM)
Human DKK1	100	6.5×10^6^	<2.9×10^−5^ e	<4.4	0.49
	28	1.4×10^7^	<2.9×10^−5^ e	<2.0	n.d.
	39	6.9×10^6^	<2.9×10^−5^ e	<4.1	0.42
Mouse DKK1	45	8.4×10^6^	<2.9×10^−5^ e	<3.4	< 0.3
Rat DKK1	100	1.0×10^7^	<2.9×10^−5^ e	<2.8	0.62
Human DKK2	n.d.^b^	n.d.	>0.3	140,000^f^	110,000
Mouse DKK2	n.d.	n.d.	>0.1	98,000^f^	n.d.
Human DKK4	2.0	1.9×10^7^	1.0×10^−4^	5.5	3.5
Mouse DKK4	30	2.7×10^7^	7.8×10^−5^	2.9	4.6
	36^c^	n.d.	n.d.	6.8	5.1

a The “% activity” was determined via titration-based assays on the Octet and used to correct all k_a_ values.

b Not determined.

c Activity determined by CFCA.

d The apparent k_a_ values determined via KinExA were 1.05×10^7^ M^−1^s^−1^ and 1.07×10^7^ M^−1^s^−1^ for human DKK1 and 1.4×10^7^ M^−1^s^−1^ and 0.80×10^7^ M^−1^s^−1^ for human DKK4 in two independent experiments each.

e The resolution limit of the assay, according to the “5% rule”.

f Measured in a kosmotropic buffer containing 150 mM (NH_4_)_2_SO_4_ instead of 150 mM NaCl.

### The active concentrations of select antigens and DS4 were determined via a calibration-free concentration analysis on the Biacore

Biacore instruments allow protein concentrations to be determined without relying upon a calibration curve, and thereby eliminate the need for a standard compound, in an analysis known as a “calibration-free concentration analysis” (CFCA) [13]. We used CFCA to analyze one lot each of human DKK1, human DKK4, and mouse DKK4. For human DKK1, the activity was determined to be 31%–39% by Biacore (Figure 4C), compared to 39% on the Octet. For human DKK4, the Biacore assay yielded an activity of 37%–39% while the Octet assay showed 45%. Mouse DKK4 showed an activity of 36%; this specific lot was not tested on the Octet but employed in our KinExA and Biacore solution affinity measurements. Purified recombinant human and mouse DKK2 were not amenable to our capture-based CFCA method because their interactions with immobilized DS4 were not sufficiently mass transport limited.

Using CFCA on amine-coupled anti-Id, we empirically verified that DS4 was fully active, thereby corroborating the assumption that underpinned our entire KinExA analysis.

### The slow dissociation of the DKK1/DS4 complex exceeded the dynamic range of a capture-based kinetics assay on the Biacore

**Figure 5:**
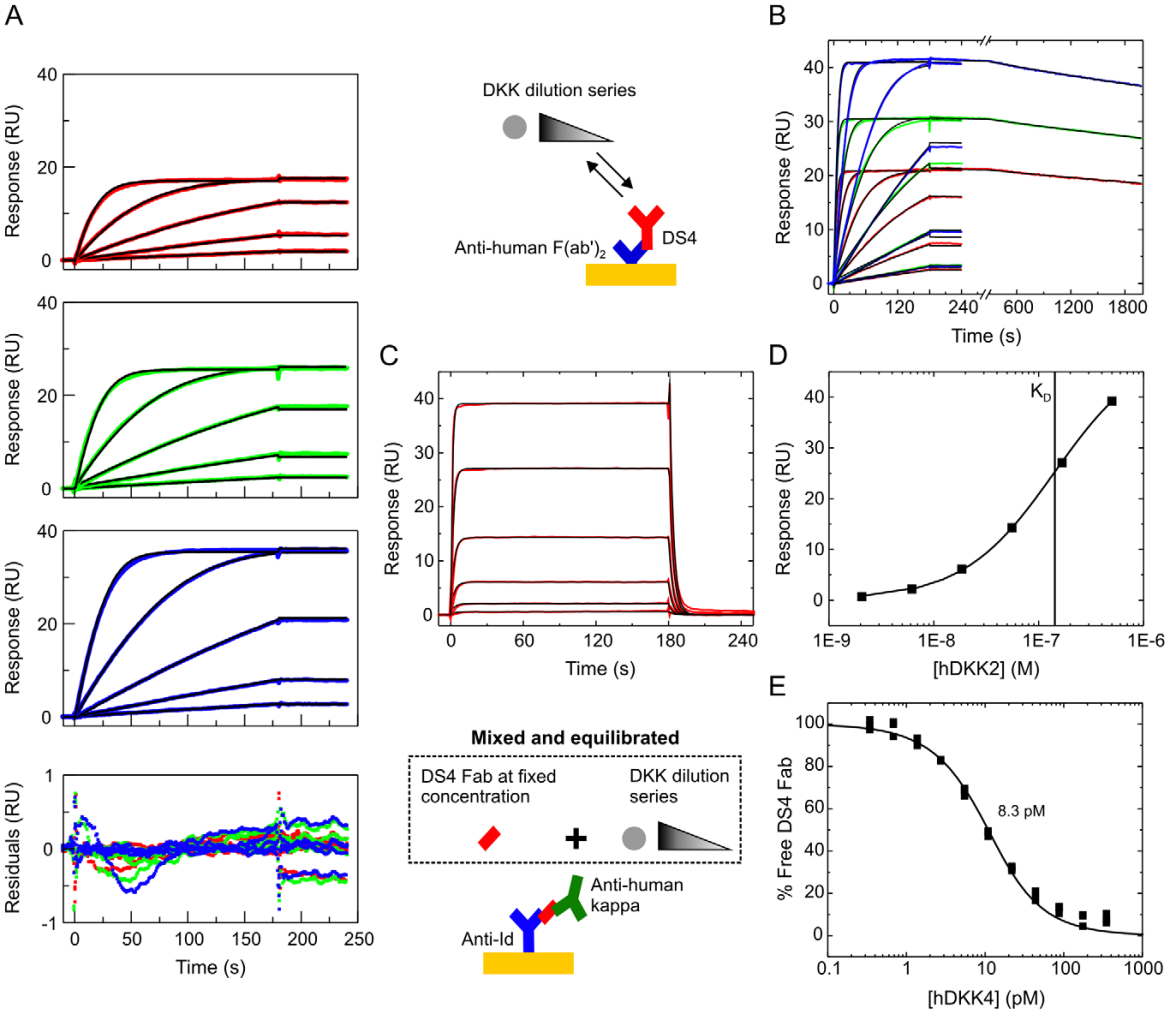
Kinetics and affinity determinations by Biacore. Binding kinetics of (A) human DKK1, (B) mouse DKK4, and (C) human DKK2. Measured data in color contrast the global fits in black. Panels A and B each show a simultaneous fit of the data obtained from three different capacity surfaces (red – low, green – medium, and blue – high; in panel A, the data are separated out by surface for clarity but were fit simultaneously). Panel C shows data from a single surface. In each case, DKK was flowed as a threefold dilution series with nominal top concentrations of (A) 13 nM, (B) 40 nM, and (C) 500 nM over immobilized DS4. Only the association phase was monitored for DKK1 because the dissociation was too slow to resolve by our capture-based assay. Panel D shows an alternate analysis of the DKK2 data, using the equilibrium binding responses. Panel E shows the inhibition of DS4 Fab with titrating levels of human DKK4 over immobilized anti-Id. See Table 2 for the extracted kinetic rate constants and affinities. The upper cartoon depicts the assay orientation used in panels A–D while the lower cartoon refers to panel E.

Preliminary studies on the Biacore revealed that the DKK proteins displayed high unspecific binding to a standard CM5 chip, so we employed an ethylenediamine-blocked anti-human Fc CM4 chip to capture DS4 in an attempt to create a surface with less residual negative charge than an ethanolamine-blocked CM5 surface (see Materials and Methods). We determined apparent association rate constants (k_a_) for all DKK1 orthologs tested (Figure 5A and Table 2) but did not observe enough decay of the binding signal during the allowed dissociation time to determine a precise dissociation rate constant (k_d_) for these interactions. Since their dissociation phase showed less than a 5% decrease in signal during the allowed 30 min, according to the “5% rule” [14], the k_d_ appeared to be <2.85×10^−5^ s^−1^. We thus calculated an upper limit for the individual affinities of the multi-species panel of DKK1, but no precise affinities. The product of the KinExA-determined k_a_ and K_D_ values gave an apparent k_d_ for human DKK1 that was about 10-fold lower than the k_d_ limit of 2.85×10^−5^ s^−1^ stated above. This implied that it would be necessary to follow the dissociation for five hours on the Biacore in order to detect a 5% signal decrease, which would require a more stable method of immobilizing DS4, such as direct amine coupling. While it was possible to couple DS4, we could not find a suitable regeneration condition for it.

For the multi-species panel of DKK1 proteins, all activity-corrected k_a_ values were within fourfold of one another and the inter-species variation was similar to the variation between experiments and antigen lots (Table 2).

### Picomolar affinities for DKK4/DS4 interactions and nanomolar affinities for DKK2/DS4 interactions were corroborated by Biacore measurements

We also determined the affinities of DS4 towards human and murine DKK4 by Biacore (Figure 5B and Table 2). In both cases, there was sufficient decay in the binding signal within the allowed 30-min dissociation time to enable a precise determination of the kinetic rate constants. While the k_d_ values were significantly higher for DKK4 than for DKK1, the k_a_ values were only slightly higher for DKK4.

The weak affinity of DS4 towards DKK2 approached the upper limit of the KinExA's dynamic range. Therefore, we also tested commercially-available purified recombinant human and mouse DKK2 proteins on the Biacore. A high unspecific binding response concealed the specific binding response when we used HBST as running buffer (a commonly used buffer in Biacore assays) in which we had studied DKK1 and DKK4. To minimize unspecific binding of our His-tagged DKK2, which had a theoretical net charge of +23 at pH 7.0, we chose a kosmotropic buffer, as used by other investigators when studying analytes with high pI values [15]. Injecting DKK2 over immobilized DS4 resulted in sensorgrams that exhibited fast association and dissociation rates (Figure 5C), so we applied an equilibrium binding model to the binding responses and determined apparent K_D_ values of 140 nM for human DKK2 and 98 nM for mouse DKK2 (Figure 5D). We verified that the binding kinetics of human DKK1 were identical in standard (HBST) and kosmotropic buffers to rule out the possibility that the kosmotropic buffer altered the DKK-binding behavior of DS4. No binding of human DKK3 to immobilized DS4 was detectable at a nominal antigen concentration of 670 nM when tested in HBST buffer.

### A Biacore solution affinity measurement corroborated the KinExA affinity determination for the mouse DKK4/DS4 interaction

While the binding affinity of a solution-based interaction can be determined via a solution competition assay on the Biacore, this method is generally unfeasible for studying very high affinity interactions because it requires the direct detection of low concentrations of the fixed binding partner, which may be below the assay's limit of detection. Accordingly, preliminary signal tests suggested that we would not be able to measure the sub-picomolar affinity of the DKK1/DS4 interaction in this way. To investigate whether the Biacore could measure a solution affinity in the single digit picomolar range, we studied the DKK4/DS4 interaction in an orientation that resembled our fixed antibody KinExA format. Thus, we amine-coupled an anti-Id onto a CM5 chip to detect free DS4 Fab in samples containing a fixed concentration of Fab equilibrated with a dilution series of mouse DKK4; the active concentrations of both binding partners had been pre-determined via CFCA. Within a 30-min association time, we did not obtain enough signal above instrument noise to reliably quantify the Fab by its real-time binding to the anti-Id surface, so we used a mouse anti-human kappa mAb as a secondary detection reagent to enhance the signal. The resulting signals were high enough to obtain an inhibition curve (Figure 5E). Fitting the data to a bimolecular binding equation using the Biacore T200 evaluation software yielded an apparent K_D_ of 6.8 pM and a standard error (for the fit) of 0.5 pM. The use of a Fab-based Biacore assay thus further corroborated our KinExA-derived K_D_ for the DKK4/DS4 interaction obtained with the full-length IgG. A calibration curve obtained with Fab alone (without any premixed DKK4) on the Biacore showed that all the response values were in a linear range, but concentrations lower than 1 pM deviated from ideal behavior as they approached instrument noise.

### Association rate constants obtained on the KinExA agreed well with the activity-corrected values determined by Biacore

**Figure 6:**
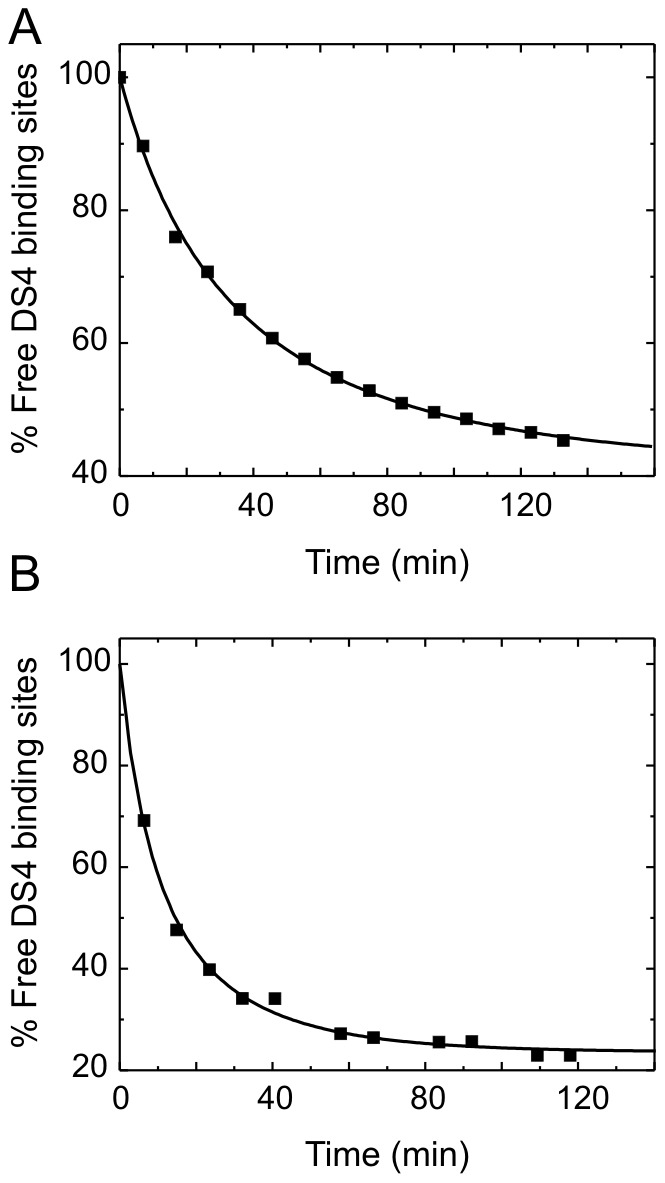
Association rate determination by KinExA. “Kinetics direct” measurements using (A) 50 pM DS4 and 30 pM human DKK1 and (B) 100 pM DS4 and 90 pM human DKK4. The apparent k_a_ values were 1.05×10^7^ M^−1^s^−1^ for human DKK1 and 1.4×10^7^ M^−1^s^−1^ for human DKK4.

It is also possible to measure the apparent association rate constant (k_a_) of an interaction via KinExA if the active concentrations of both binding partners are known. Using the “kinetics direct” method [5] as defined in the KinExA software, we determined apparent k_a_ values for human DKK1 (Figure 6A) and human DKK4 (Figure 6B) binding to DS4. In these experiments, we input the antigen's active concentration that had been determined previously in the KinExA-based equilibrium measurements described above. We obtained excellent agreement between KinExA- and Biacore-derived k_a_ values (Table 2).

## Discussion

### The KinExA's dynamic range allows discrimination between picomolar and femtomolar binders and is limited mainly by practical requirements of large volumes and/or high concentrations

During the selection and maturation of therapeutic antibodies, very high on-target binding affinities can be achieved, with K_D_ values in the picomolar [16] and subpicomolar [17] range. Characterizing these tight interactions with label-free biosensors can be challenging. The KinExA, however, enables the discrimination between picomolar and femtomolar binding affinities and provides exquisite sensitivity in resolving K_D_ values in the single digit picomolar range. Our results demonstrate how we exploited the antigen-binding activities of an anti-DKK1 mAb to highlight the wide dynamic range of the KinExA technology by measuring affinities spanning 160 fM to 120 nM for DKK1 and DKK2, respectively. These are among the tightest [12], [17]–[19] and weakest [11], [20] antigen/antibody affinities reported using the KinExA. Determination of femtomolar K_D_ values exceeds the dynamic range of a capture-based kinetics assay on SPR-based biosensors such as the Biacore because the dissociation of the antigen/antibody complex being studied approaches, or is slower than, the dissociation of the antibody from its immobilized capture partner. Studying femtomolar binders via KinExA often required the fixed component to be at a low concentration (at or below 1 pM binding sites), which introduced some practical challenges. In studying DKK1 in the fixed antibody orientation, we required high sample volumes (up to 60 mL for duplicate analysis cycles) to allow sufficient capture on the bead-immobilized binding partner, which resulted in up to 35 hours run time per experiment that typically analyzed a titration series in duplicate cycles. Moreover, judging from the association rate and apparent affinity of DKK1, it would possibly have taken over a week to equilibrate the samples containing 0.3 pM DS4 and the lowest DKK1 concentration of the titration series (i.e., 15 fM). Since antigen degradation may occur during prolonged incubation at room temperature, we compromised by incubating our samples for no longer than three days. This means that when titrating antigen into 0.3 pM DS4, only the samples with picomolar or higher DKK1 concentrations were at or approached equilibrium; the K_D_ of the interaction is thus probably slightly lower (i.e., the affinity is likely tighter) than determined in this experiment. Since an “n-curve” analysis is based upon the assumption that the interacting partners have the same activity in all experiments included in the global fit, we routinely verified this by analyzing stoichiometry-controlled curves, or in most cases sweet spot curves, at the beginning and end of a series of experiments. Alternatively, we aimed for a K_D_-controlled curve (as in our DKK2 work, Figure 3C) or a sweet spot curve (as in our DKK4 work, Figure 2A) in a single experiment.

The main limitation in characterizing low affinity interactions on the KinExA is the need for high concentrations of at least one of the binding partners, which may render an assay impractical and/or prohibitively expensive to implement in a routine manner. For example, to determine precise K_D_ values above 100 nM and k_d_ values near 10^−1^ s^−1^, we needed milligram quantities of antibody in order to saturate the antigen, as we demonstrated in our analysis of DKK2 in the fixed antigen assay orientation. Since the bead was coated with the same antibody as the one being studied in solution, the efficiency of the DKK2-capture and its subsequent detection was poor. In contrast, we were able to determine a steady-state binding affinity for DKK2 with much less protein (0.1 µg antibody and 2 µg antigen) using the Biacore capture-based kinetics assay.

### The choice of assay orientation employed in our KinExA-based DKK/DS4 work was driven mainly by the available concentrations of the respective antigens

In studying the interactions of DS4 with recombinant DKK1 and DKK4, which had apparent K_D_ values of approximately 0.4 pM and 5 pM, we confirmed that the fixed antigen and fixed antibody assay orientations yielded equivalent results (Figure 1 and Table 1), consistent with observations from other investigators [10]. Therefore, practical considerations can dictate the choice of the assay orientation employed. In this study, we used the fixed antibody orientation to study antigens that were available at high concentrations, and the fixed antigen orientation when antigens were available only at low concentrations.

An appealing feature of the fixed antibody assay orientation is that it allows the comparison of different antigens binding to the same antibody without changing the assay format in terms of the bead-immobilized partner and the detection strategy. In addition, by substituting the antigen with an anti-Id as the bead-immobilized partner, we significantly reduced our consumption of antigen and bypassed the need for purified antigen altogether when we applied our anti-Id platform to the study of unpurified antigen (as in our rhesus DKK4 work, Figure 3A). In the case of DKK proteins, bead-immobilizing the purified commercially available antigen would have cost $300 per experiment. In contrast, anti-Id reagents that only bind the free antibody, once generated, are useful in assays where unspecific antigen-antigen interactions occur or when the antigen's activity is compromised or lost upon its immobilization to the bead.

The fixed antigen assay orientation requires more antigen-specific assay development, especially in terms of the detection strategy used. However, the ability to float both the apparent K_D_ and the apparent active concentration of one of the binding partners (assuming that the other is 100% active and that the binding stoichiometry of the interaction is known) along with the double specificity required to capture and detect one of the binding partners renders the KinExA particularly amenable to studying native antigens that may only be available at a low, constant, but unknown concentration, such as in complex media [10]. Thereby, the KinExA bypasses the need for purifying the studied antigens, which not only saves time but avoids the risk of altering an antigen's native conformation or activity. In customizing the detection antibodies to suit each studied antigen in our fixed antigen KinExA experiments, we found that mixing an antigen-specific antibody with the fluorescent probe (DyLight-labeled antibody or DyLight-streptavidin) reduced the detection signal about twofold when compared to injecting the two reagents consecutively. Taking into account sensitivity, specificity, and assay run time, we obtained optimum results for DKK1 and DKK4 detection with directly DyLight-labeled sandwiching antibodies.

### The use of a sweet spot concentration facilitates the analysis of tight binders and precious samples

Our results show that fixing one binding partner four- to sixfold above K_D_ typically resulted in a titration curve that contained sufficient information to estimate with good accuracy and precision both the apparent K_D_ and the apparent active concentration of the unknown binding partner. This highly informative sweet spot curve lies in the transition range between K_D_-controlled and stoichiometry-controlled curves, as graphed in the landscape plot in Figure 2E. The broadness of the sweet spot ultimately depends on data quality. Designing an experiment that will yield a sweet spot curve often relies on *a priori* knowledge of both the apparent K_D_ and the apparent active concentration of the fixed binding partner. Another potential drawback of using a sweet spot analysis is that interactions deviating from a simple bimolecular binding model (e.g., due to sample heterogeneity or cooperative binding) will be overlooked because the concentration-to-K_D_ ratio in the analysis will adjust to fit the curve steepness thus causing an error in the result and masking the perturbation. An n-curve analysis, however, will correctly identify this perturbation because the curve steepness will not match the spacing between the curves.

Nevertheless, in exploring inter-species variation within a given DKK protein (e.g., mouse, human, and rhesus forms of DKK4), comparing recombinant and native forms of a given DKK, or comparing different assay orientations (i.e., fixed antigen and fixed antibody) within the KinExA platform, we typically had a good *a priori* estimate of the apparent K_D_. Thus, the use of a sweet spot assay increased the information content of an experiment and saved a significant amount of sample. While an n-curve analysis can often increase the precision of a K_D_ determination compared with that from a single experiment, it relies on the assumption that the antigen's activity remains constant across a series of experiments. This may not be the case if there is degradation of the antigen from one experiment to the next, or if there is significant lot-to-lot variability as we observed for DKK preparations; a sweet spot analysis circumvented this problem. Furthermore, working several fold above K_D_ avoided a number of the practical disadvantages related to working at low picomolar concentrations, such as high sample volumes and long equilibration and run times which might result in sample deterioration. Also, when determining a K_D_ from a purely K_D_-controlled curve, the active concentration of the titrated binding partner should be accurately known. In contrast, K_D_ determination with a sweet spot assay requires only one concentration to be known, which can be that of either the fixed or titrated component.

### Tailoring the KinExA method enables the study of a broad range of affinities as well as sample qualities and quantities

Our work has demonstrated that the KinExA is a highly versatile biophysical tool that can be used to study interactions that span a broad range of affinities and can be tailored to accommodate the available sample quality and quantity. We were thus able to verify the affinity of DS4 towards its native, unrefined target protein. Furthermore, the affinity measurements were in excellent agreement with those obtained on Biacore, for interactions that fell within the dynamic range of both methods (i.e., K_D_ values spanning single digit pM to mid nM).

## Materials and Methods

### General Methods

A KinExA 3000 biosensor with standard flow cells and polymethylmethacrylate (PMMA) beads was purchased from Sapidyne, Inc (Boise, ID). A Biacore T200 biosensor equipped with CM4 and CM5 research-grade sensor chips was purchased from GE Healthcare (Piscataway, NJ). An Octet QK384 biosensor equipped with amine-reactive and anti-murine-Fv sensor tips was purchased from ForteBio (Menlo Park, CA). Recombinant full-length DS4 IgG and DS4 Fab were prepared in-house from suspension-adapted HEK293 cell (“293F” cells, Invitrogen, Carlsbad, CA) conditioned media, which was collected five days after transient transfection with CMV-based expression vectors encoding both the light and heavy chains. Purification of DS4 IgG and DS4 Fab was performed via protein A or Ni-NTA affinity chromatography, respectively. Murine anti-idiotype mAb raised against DS4 and murine mAb raised against human DKK4 were generated in-house via conventional hybridoma technology. Purified recombinant human DKK1, DKK2, DKK3 and DKK4, mouse DKK1, DKK2, and DKK4, rat DKK1, mouse anti-human DKK1 mAb (clone 141119), biotinylated polyclonal antibody raised against human DKK4, and mouse anti-polyhistidine mAb (clone AD1.1.10) were obtained from R&D Systems (Minneapolis, MN). Mouse anti-human kappa mAb (clone SB81.a) was purchased from Southern Biotech (Birmingham, AL). DyLight 649 streptavidin and DyLight 649 AffiniPure polyclonals produced in goat against human IgG (H+L) or mouse IgG (H+L) with minimum cross-reactivity to other species were purchased from Jackson ImmunoResearch Laboratories, Inc. (West Grove, PA). DyLight 649 microscale antibody labeling kit and IgG elution buffer pH 2.8 were purchased from Pierce Biotechnology (Rockford, IL). Anti-DKK1 and anti-DKK4 antibodies were DyLight-coupled following the manufacturer's protocol. Polyclonal goat F(ab′)_2_ anti-human Fc was purchased from Cappel (MP Biomedicals, Solon, OH). Quantification of select antigens by sandwich ELISA was performed using DS4 as the capture antibody and a suitable sandwiching antibody, as used for detection in KinExA experiments performed in the fixed antigen orientation (see below).

Theoretical pI values and charges at neutral pH were calculated from the primary amino acid sequence using Vector NTI Advance 9 (Invitrogen). All concentrations were calculated as binding site concentrations. Where applicable, “nominal” concentrations were determined via light absorption at 280 nm; for BSA-containing antigen preparations the manufacturer's information was used instead.

### Preparation of in-house antigens

The complete coding regions of cyno DKK2 and rhesus DKK4 were obtained by PCR from cyno liver (DKK2) or rhesus testis (DKK4) cDNAs (BioChain, Hayward, CA), using minimally degenerate 5′ and 3′ primers, which were designed based on the available rhesus GenBank nucleotide sequences (XM_001085254 and XM_001097758). Amplified fragments were sequence verified, and a 10-His epitope tag and stop codon were added in-frame at the extreme C terminus by PCR. The resulting DNA fragments were individually cloned into a CMV-based expression vector. The cDNA containing the complete coding region of human DKK2 was obtained from Origene (Rockville, MD). It was sequence verified, and re-cloned into a CMV-based expression vector which added an in-frame 10-His tag to the C-terminus. To obtain conditioned media containing human DKK2, cyno DKK2 or rhesus DKK4, 293F cells were grown in defined serum-free media (Freestyle Expression Media, Invitrogen) and transiently transfected with an expression construct for the respective antigen using 293Fectin (Invitrogen) according to the manufacturer's protocol. Twenty four hours after transfection, heparin (Sigma-Aldrich, St. Louis, MO) was added at 0.1 g/L to enhance recovery of DKK in the conditioned media [21]. Cells were allowed to condition the media for five days, after which time the media was collected.

Conditioned media from Hep3B cells expressing native human DKK1 was produced by growing Hep3B cells (ATCC, Manassas, VA) in DMEM media supplemented with 10% heat inactivated fetal bovine serum, 100 U/mL penicillin and 100 µg/mL streptomycin at 37°C in an atmosphere of 5% CO_2_. The cells were plated at a density of 2×10^5^/cm^2^ and media was collected after three days.

### KinExA measurements

All KinExA experiments were performed at room temperature (23°C) using PBS pH 7.4 with 0.01% (v/v) Tween-20 as running buffer. Samples were prepared in running buffer supplemented with 1 mg/mL BSA (“sample buffer”), unless stated otherwise. A flow rate of 0.25 mL/min or 0.4 mL/min was used, unless stated otherwise. PMMA beads were adsorption-coated with a capture antibody (murine anti-Id mAb or DS4 for affinity determinations in the fixed antibody and fixed antigen orientations, respectively) to serve as the solid phase. Per experiment, 200 mg beads were prepared at room temperature by incubating them with 30 µg capture antibody in 1 mL PBS for 2 h with rocking and blocking them in 10 mg/mL BSA in PBS for a further 1 h with rocking. Blocked beads were either used immediately or stored for up to one week at 4°C.

In the fixed antibody orientation, DS4 was prepared at a fixed concentration, which was optimized per experiment, and titrated with a twelve-membered twofold serial dilution of the DKK to be tested. A sample of DS4 without any titrated DKK established the 100% signal, i.e., the signal without any inhibition. Samples were incubated for up to 72 h at room temperature before passing them through a flow cell that contained bead-immobilized murine anti-Id mAb. Captured DS4 was detected with 1 µg/mL DyLight-labeled polyclonal anti-human antibody.

Affinity determinations in the fixed antigen orientation were performed by titrating DS4 as a twofold dilution series into a fixed concentration (or in the case of crude antigens, a fixed dilution) of the antigen, equilibrating the samples at room temperature, and passing them through a flow cell that contained beads coated with DS4. Detection of the bead-captured antigens was tailored per antigen using 0.5 µg/mL or 1 µg/mL of a “sandwiching” antibody (one whose epitope did not overlap with that of the bead-immobilized mAb) followed by a DyLight-labeled reagent. Thus, human DKK1 was detected in two steps using mouse anti-human DKK1 mAb followed by DyLight 649 AffiniPure goat anti-mouse IgG, or in a single step using DyLight-labeled mouse anti-human DKK1 mAb. Recombinant His-tagged mouse DKK1, human DKK2, and cyno DKK2 were each detected using mouse anti-polyhistidine mAb followed by DyLight 649 AffiniPure goat anti-mouse IgG. Human DKK4 was detected using biotinylated goat polyclonal anti-human DKK4 antibody followed by DyLight-conjugated streptavidin or by DyLight-labeled mouse anti-human DKK4 mAb.

Association rate constants of DS4 binding human DKK1 or human DKK4 were obtained via the “kinetics direct” method predefined in the KinExA software [5]. Thus, DS4 and DKK were mixed and sample volumes of 0.25 mL (or in some experiments, 0.5 mL) were drawn in regular time intervals through the flow cell where free DS4 binding sites were captured on anti-Id-coated beads and detected with DyLight-labeled polyclonal anti-human IgG.

KinExA data were analyzed with the KinExA Pro software versions 2.0.1.12, 2.0.1.26, and 2.1.1.28 provided by Sapidyne. To determine the apparent K_D_ and the apparent active concentration of the antigen, the “affinity, unknown ligand” or “affinity, standard” models were used to analyze data obtained in the fixed antibody or fixed antigen orientations, respectively. The “drift correction” option was used where appropriate. DS4 was assumed to be 100% active (validation of this assumption is described in the results). For a given interaction, multiple curves obtained in independent experiments were analyzed using the “n-curve analysis” tool to obtain global best fit values for both the apparent K_D_ and the apparent antigen activity. The software reports each best fit value along with a 95% confidence interval (Sapidyne Technote TN206R0). The apparent k_a_ values were determined by fitting the decrease of free antibody binding sites as a function of time to a bimolecular binding equation using the “kinetics direct” analysis tool.

### Titration-based active concentration measurements on the Octet biosensor

All Octet experiments were conducted at 25°C in a running buffer of PBS pH 7.4, 0.05% (v/v) Tween-20 and 1 mg/mL BSA, and sample plates were agitated at 1000 rpm. An anti-Id mAb was coupled onto amine-reactive tips that were first activated with a freshly prepared mixture of 40 mM EDC and 10 mM sulfo-NHS (final concentrations) in 0.1 M MES buffer pH 5.0. Excess esters were blocked with 1 M ethanolamine HCl pH 8.5. DKK1 or DKK4 were titrated into 1 nM or 2 nM DS4 binding sites using a 1.2-fold dilution series. These mixtures were allowed to bind the coupled anti-Id for 30 min to detect free DS4 binding sites. The binding of DS4 without any premixed DKK established the 100% signal. We confirmed that neither DS4 nor DKK bound unspecifically to the unmodified tips or the coupled anti-Id respectively. Some samples were analyzed on duplicate tips to verify that the assay was reproducible between tips.

Octet data were exported into Scrubber v.2.0a (BioLogic Software Pty Ltd, Australia) for data processing and analysis. The binding responses obtained for buffer were subtracted from those obtained for the DKK/DS4 premixes over the coupled anti-Id. These “blank-subtracted” binding responses were plotted against the “nominal” antigen concentration to construct an inhibition curve. The equivalence point of the stoichiometry-controlled titration is thereby defined as the lowest nominal antigen concentration that fully inhibits binding of a “known” concentration of DS4 binding sites. The ratio of the active concentration/nominal concentration was expressed as a percentage to give the antigen's activity.

### Calibration-free concentration analysis (CFCA) on the Biacore

All CFCA experiments were conducted at 25°C on a Biacore equipped with a CM5 sensor chip, as per the manufacturer's recommendations. The running buffer was HBST (10 mM HEPES pH 7.4, 150 mM NaCl, 0.05% (v/v) Tween-20) for the immobilization and HBST supplemented with 1 mg/mL BSA was used for the interaction analysis. To prepare reference and reaction surfaces appropriate for the CFCA of antigens, flow cells 1 and 2 were activated with a freshly prepared mixture of 0.2 M EDC and 0.05 M NHS (final concentrations) for 7 min, 60 µg/mL polyclonal goat F(ab′)_2_ anti-human Fc in acetate pH 5.0 was coupled for 7 min, and excess reactive esters were blocked with 0.1 M ethylenediamine in 150 mM sodium borate pH 8.5 for 7 min. The use of ethylenediamine instead of ethanolamine (the standard blocking reagent for amine-coupling) was intended to yield a surface with less negative charge than that blocked with ethanolamine. Final immobilization levels of the capture reagent were approximately 7,000 RU. The running buffer (HBST) was then supplemented with 1 mg/mL BSA and DS4 was captured on flow cell 2 at a typical level of 790 RU, leaving flow cell 1 blank (naked capture reagent) to provide a reference surface. Each antigen tested (human DKK1, DKK2 and DKK4) was serially diluted to typical concentrations of 1, 0.1, and 0.01 µg/mL and injected in duplicate at flow rates of 5 µL/min and 100 µL/min for 36 sec over flow cells 1 and 2. The capture surfaces were regenerated with two 30-sec injections of 75 mM phosphoric acid. Double-referenced binding responses were analyzed using the CFCA tool in the Biacore T200 software.

To prepare appropriate reference and reaction surfaces for the CFCA of DS4 (IgG and Fab), an irrelevant IgG or an anti-DS4 anti-Id were immobilized on flow cells 1 and 2 respectively of a CM5 chip via amine-coupling using a standard blocking reagent (1 M ethanolamine HCl pH 8.5). In some experiments, an activated and blocked surface served as the reference surface. The CFCA was performed as described above for antigens, except that surfaces were regenerated with a 2∶1 (v/v) blend of Pierce IgG elution buffer/4 M NaCl when performing CFCA of the Fab.

### Kinetic and affinity measurements on the Biacore

Kinetic assays were conducted at 25°C using CM4 sensor chips. Ethylenediamine-blocked anti-human Fc capture surfaces were prepared on all four flow cells using a procedure similar to that described above. This resulted in final immobilization levels of approximately 4000 RU per channel. Interaction analyses of DS4 with DKK1, DKK3, and DKK4 antigens were performed in HBST running buffer supplemented with 1 mg/mL BSA. DKK2/DS4 interactions were analyzed in a kosmotropic running buffer (10 mM HEPES pH 7.4, 150 mM ammonium sulfate, 0.05% (v/v) Tween-20, and 1 mg/mL BSA). The reaction surfaces were prepared by capturing DS4 onto different flow cells at typical levels of 45, 70, and 95 RU. A flow cell without any captured DS4 (bare capture surface) served as a reference surface. DKK1, DKK2, and DKK4 samples from different species were each prepared as a threefold dilution series with a top concentration of 13 nM (DKK1), 40 nM (DKK4) or 500 nM (DKK2) and injected at 30 µL/min typically for 3 min using a variable dissociation time [14]. The capture surfaces were regenerated with two 30-sec injections of 75 mM phosphoric acid. No regeneration of the surface was performed when analyzing human DKK2 because it completely dissociated within a three minute- dissociation phase. Some antigens were analyzed in duplicate independent runs to verify that the K_D_ determination was reproducible. Human DKK3 was injected at 67 nM and 670 nM over DS4 captured at 110 RU in a manual run using HBST running buffer.

Kinetic data obtained on the Biacore were processed and analyzed in Biacore T200 evaluation software version 1.0. Double-referenced data [22] from three different capacity reaction surfaces were fit globally to a simple 1∶1 Langmuir model with mass transport to extract the apparent association (k_a_) and dissociation (k_d_) kinetic rate constants, whose ratio gave the apparent equilibrium dissociation constant (K_D_ = k_d_/k_a_). The titration results from the Octet solution competition assays (described above) were used to adjust the “nominal” antigen concentrations to “active” antigen concentrations, and thus correct the apparent k_a_ and K_D_ values accordingly. The K_D_ for the DKK2/DS4 interaction was determined using an equilibrium binding model.

### Solution affinity measurement on the Biacore

Prior to determining the solution affinity of mouse DKK4 binding to DS4 Fab, the active concentrations of both reagents were empirically determined via CFCA, as described above. To perform a solution affinity measurement, the reaction surface was prepared by amine-coupling an anti-Id to a CM5 chip and blocking excess reactive esters with ethylenediamine. An activated and ethylenediamine-blocked surface served as a reference surface. Mouse DKK4 was titrated as a twelve-membered, twofold dilution series with a top concentration of 700 pM into 8.3 pM DS4 Fab and these samples were allowed to equilibrate at 25°C. Additionally, a calibration curve was prepared using a twofold serial dilution of the Fab alone. All samples were injected in triplicate over the reference and reaction surfaces for 30 min at 10 uL/min, followed by a two-minute injection of 15 ug/mL mouse anti-human kappa mAb to enhance the signal. Surfaces were regenerated with two 30-sec injections of a 2∶1 (v/v) blend of Pierce IgG elution buffer/4 M NaCl.

Single-referenced data from the enhancement step were obtained by subtracting the reference surface responses from the reaction surface responses and analyzed with the solution affinity model in the Biacore T200 evaluation software. Thus, the free Fab concentration in each sample was determined via the calibration curve and the K_D_ fitted to a bimolecular binding equation. For Figure 5E, the y-axis was converted to the percentage of free DS4 Fab in order to resemble the KinExA plots.
